# Predator Experience Shapes Behaviour: Comparing Stone Wētā (*Hemideina maori*) Populations With and Without Weka ( *Gallirallus australis hectori* )

**DOI:** 10.1002/ece3.73907

**Published:** 2026-06-29

**Authors:** Sheri Johnson, Luke Thompson, Hamish Doogan, Priscilla Wehi

**Affiliations:** ^1^ Department of Zoology University of Otago Dunedin New Zealand; ^2^ Centre for Sustainability University of Otago Dunedin New Zealand

**Keywords:** antipredator behaviour, behavioural plasticity, introduced predator, island translocation, predation, predator‐free

## Abstract

Antipredator behaviour reflects both evolutionary history and individual experience, yet how populations respond to changes in predator exposure remains poorly understood, particularly for large invertebrates. We examined antipredator behaviour in two populations of stone wētā (*Hemideina maori* Pictet & Saussure, 1891) inhabiting weka‐free Mou Tapu and nearby Mou Waho, where weka (*Gallirallus australis hectori—*a large flightless rail) were translocated in 2004 and persist. Wētā were collected from both islands and assayed under controlled laboratory conditions to quantify refuge use, locomotor activity, exploratory behaviour, and defensive responses to simulated attack. Morphological traits did not differ between islands, aside from a weak trend toward longer hind femora in Mou Waho individuals. Refuge‐seeking behaviour and open‐field activity did not vary between populations, nor did exploratory tendencies. However, predator‐experienced Mou Waho wētā were substantially more defensive: 84% responded aggressively to the first simulated attack compared with 58% of Mou Tapu individuals, and larger wētā showed stronger defensive responses irrespective of origin. Defensive behaviour types (fending, fleeing, rasping) were conserved across populations, suggesting that differing recent exposure to weka altered the threshold at which antipredator behaviours were deployed rather than the underlying behavioural repertoire itself. Field observations revealed striking microhabitat restriction on Mou Waho, where wētā were found only beneath large summit rocks, consistent with sustained predation pressure. Our findings demonstrate that predator experience selectively enhances reactive components of antipredator behaviour without altering proactive behaviours such as refuge‐seeking or activity. These results highlight the importance of behavioural assessments in conservation planning, especially when predator‐naïve populations may face re‐exposure to native predators through restoration or translocation programmes.

## Graphical abstract

Map of Lake Wānaka showing the locations of Mou Tapu and Mou Waho, the two islands where Hemideina maori were sampled, with an inset image of a female *H. maori*. Mou Tapu is free of introduced mammalian predators and weka ( *Gallirallus australis hectori* ), whereas Mou Waho hosts an introduced population of weka.

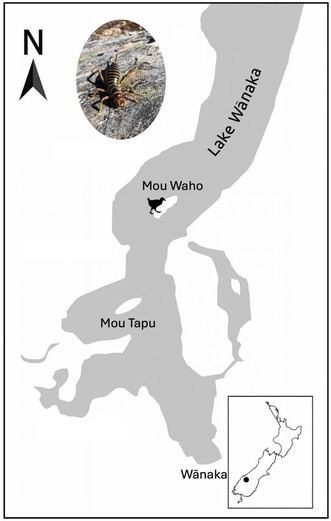

## 1 Introduction

Understanding how prey respond to predators is central to predicting ecological resilience, particularly in ecosystems undergoing rapid change or active restoration. Climate perturbation is one important driver of ecosystem change, where the establishment of introduced species, or spread of native species outside of their historical distributions, may lead, for example, to prey encountering predators they have not previously experienced (Gaynor et al. 2024; Laws 2017; Weiskopf et al. 2020). Similarly, ecological restoration also relies on methods such as translocations that can result in novel species interactions, where there is no prior history of predator–prey encounter within a population (Armstrong et al. 2015; Carthey and Banks 2014; Sih et al. 2010; Whitwell et al. 2012). On the other hand, eradication of established introduced predators can result in predator‐naïve prey that may be poorly prepared for future predator incursions (Bannister et al. 2018; Blumstein 2002; Carthey and Banks 2014; Cox and Lima 2006; Muralidhar et al. 2019; Whitwell et al. 2012).

Antipredator behaviour encompasses a suite of proactive and reactive traits, including refuge selection, vigilance, reduced movement, and defensive displays, that influence individual survival and population persistence. These behaviours are shaped both by evolutionary history and by direct experience with predators, and can diverge markedly between predator‐naïve and predator‐exposed populations (Bannister et al. 2018; Blumstein 2002; Cox and Lima 2006; Muralidhar et al. 2019; Whitwell et al. 2012). In environments where predators are absent, prey may exhibit behavioural naïvety, responding weakly or inappropriately to predation threats. Comparative studies across taxa show that some populations fail to recognise cues from novel or non‐co‐evolved predators (Muralidhar et al. 2019; Whitwell et al. 2012), while others generalise antipredator responses across predator types, leading to striking differences in vulnerability (Ünlü et al. 2020). Similar patterns have been documented in both terrestrial and aquatic systems, where populations with a history of predation respond more flexibly to predator‐derived cues than populations from predator‐free environments (Whitwell et al. 2012; Wisenden et al. 1997). Such divergence is especially pronounced on islands, where the loss or absence of predators may be associated with behavioural relaxation, or conversely vulnerability to novel predators (Blumstein and Daniel 2005; Sih et al. 2010; Whitwell et al. 2012).

New Zealand provides an exceptional context for studying these dynamics. Its native fauna evolved largely without mammalian predators (Gibbs 2009) but in the presence of avian predators, many of which exert strong selective pressure on invertebrates (Gibbs 2010). With an active conservation programme that includes species translocations and eradications, numerous offshore islands and lake islands serve as conservation sanctuaries (Innes et al. 2019; Jones et al. 2016; Russell et al. 2015), creating natural laboratories in which predator regimes differ sharply over small spatial scales. These interventions have repeatedly demonstrated that antipredator behaviour can be both lost and regained over relatively short timeframes. For example, translocations into predator‐free sanctuaries have been associated with rapid relaxation of antipredator behaviours in birds (Muralidhar et al. 2019), while eradication of invasive mammals has led to measurable changes in refuge use, activity, and foraging behaviour in large invertebrates such as tree wētā (Kelly et al. 2023; Rufaut and Gibbs 2003; Watts et al. 2011). Although these shifts may reflect adaptive responses to reduced predation risk, they also raise concern: if predators reinvade, or if native predators are introduced into systems containing naïve prey, behavioural mismatches could lead to heightened mortality. Understanding whether endemic species retain, lose, or generalise antipredator responses therefore has direct implications for the long‐term success of conservation and restoration efforts.

We investigated antipredator responses of an endemic New Zealand insect, the stone wētā (*Hemideina maori*), using two island populations that differ in exposure to an avian predator. Both islands are free of introduced mammalian predators, but Mou Waho supports a translocated population of weka (*Gallirallus australis hectori*), an opportunistic omnivorous bird capable of exerting strong predation pressure on large invertebrates (Beauchamp 1998; Carpenter et al. 2021; Carroll 1963), whereas nearby Mou Tapu remains weka‐free. This contrast provides a rare opportunity to examine how exposure to a native avian predator influences antipredator behaviour in an invertebrate system, without the confounding effects of introduced mammalian predation.

We assessed locomotor activity, refuge‐seeking, exploratory tendencies, and defensive responses under controlled laboratory conditions, as these behaviours capture key axes of antipredator strategy related to detection, avoidance, and defence (Field and Glasgow 2001; Kelly et al. 2023; Parli et al. 2020; Thompson et al. 2024). Based on predator‐exposure theory, we predicted that wētā from the weka‐free island (Mou Tapu) would exhibit weaker defensive aggression and more active and exploratory behaviour, whereas wētā exposed to weka predation (Mou Waho) would show stronger and more immediate defensive responses, accompanied by reduced activity and exploration. By isolating the behavioural effects of an endemic avian predator, this study provides new insight into how changes in predator presence shape invertebrate behaviour, with direct relevance for conservation management, species translocations, and predator restoration planning.

## 2 Methods

### 2.1 Study Species and Study Sites

**FIGURE 1:**
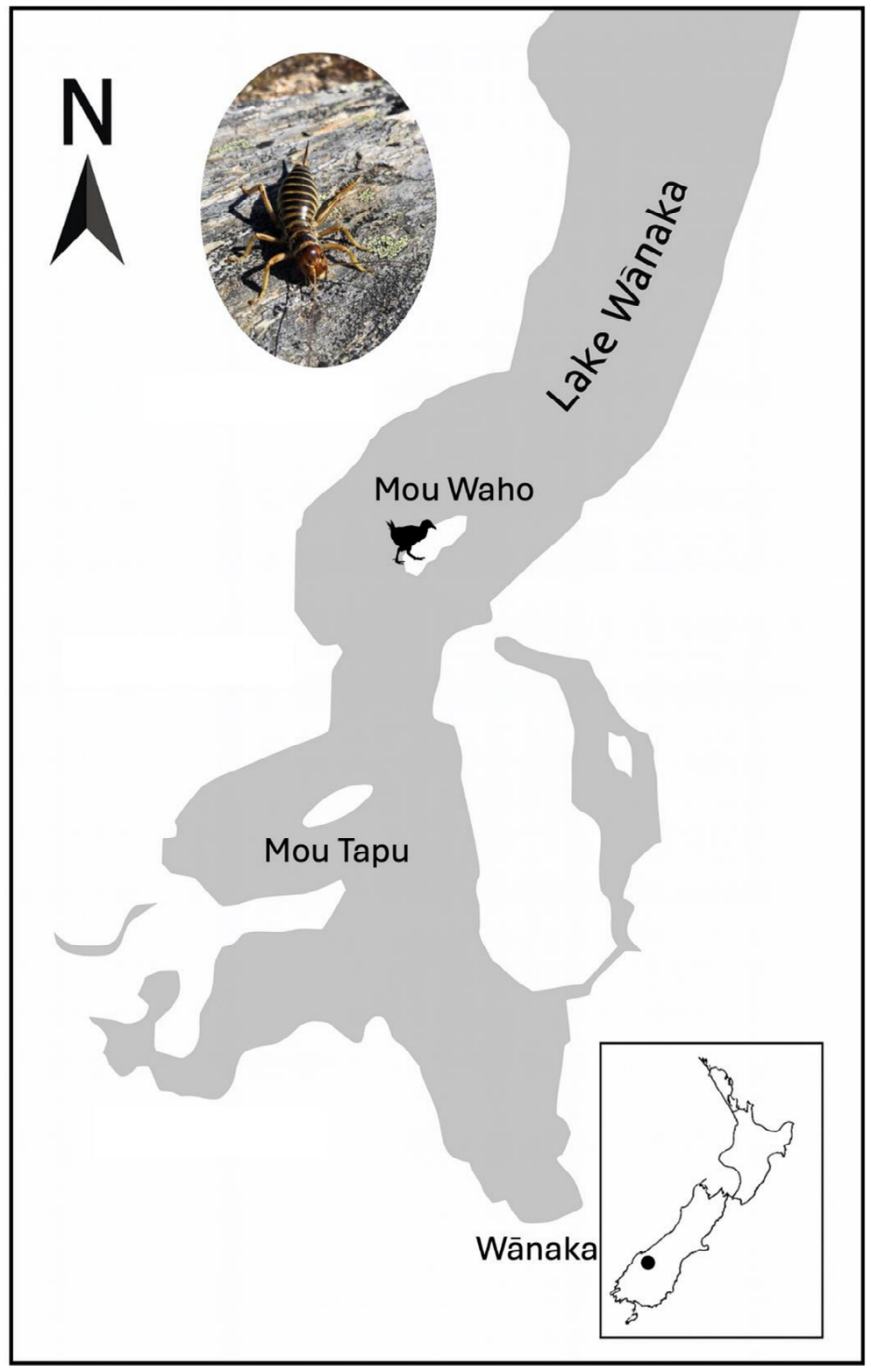
Map of Lake Wānaka showing the locations of Mou Tapu and Mou Waho, the two islands where *Hemideina maori* were sampled, with an inset image of a female *H. maori*. Mou Tapu is free of introduced mammalian and weka ( *Gallirallus australis hectori* ), whereas Mou Waho hosts an introduced population of weka. Mou Waho (118 ha) and Mou Tapu (126 ha) are small islands located within Lake Wānaka, separated by approximately ~2 km.

*Hemideina maori*, hereafter referred to as wētā, are long‐lived, flightless, nocturnal orthopterans that inhabit rock tors and rock‐under‐rock spaces in the South Island of New Zealand. They are most commonly found at elevations of 1100–1500 m (Leisnham and Jamieson 2002), but also occur at low elevations, including on Mou Waho and Mou Tapu islands (~300 m) in Lake Wānaka (Figure 1; King 2015). Populations consist primarily of yellow to intermediate colour morphs, with melanic individuals present in some areas, including both study islands (King 2015; King et al. 2003). Adults are relatively large‐bodied insects, with a mean body mass of 3.95 ± 0.53 g SD (standard deviation) and a mean right hind femur length of 14.51 ± 1.12 mm SD.

These wētā exhibit a suite of antipredator behaviours, including stridulation (rasping femur spines against the abdomen), mandible gaping, and hind‐leg raising (Field 2001; Field and Glasgow 2001; Thompson et al. 2024). The species is sexually dimorphic: males possess enlarged mandibles used in both intrasexual competition and defence (Koning and Jamieson 2001).

Buff weka ( *Gallirallus australis hectori* ) are large, flightless, diurnal rails endemic to New Zealand and are known as opportunistic omnivores that prey on a wide range of invertebrates, including large‐bodied insects (Carpenter et al. 2021; Carroll 1963). Buff weka were once common on the eastern South Island—the subspecies went extinct on the mainland by 1924 (Coleman et al. 1983; King 2017), but in 1905 a few were translocated to Rēkohu | Wharekauri | Chatham Islands where they are now abundant. They have since been translocated to the islands of Lake Wakatipu and Lake Wānaka, including Mou Waho Island in 2004, where the population is currently estimated at approximately 200 individuals. Weka are absent from neighbouring Mou Tapu Island.

Both islands lie within the historical range of weka, and would have been accessible to them given their known swimming ability (Riddell and Riddell 2012), making historical presence on both islands likely, although not directly documented. As such, wētā at both sites are likely to share an evolutionary history with weka, but differ in recent exposure.

In these systems, potential native predators of *H. maori* are likely limited to geckos and nocturnal avian predators (Field and Glasgow 2001), such as ruru (morepork). In contrast, weka are persistent ground‐foraging predators capable of directly encountering wētā during refuge emergence or movement. Weka can forage throughout the day and night, with crepuscular peaks of activity (Carpenter et al. 2021; Lamb et al. 2021).

Both Mou Tapu and Mou Waho are free of introduced mammalian predators. Mou Waho experienced a mouse incursion in 1995 that was quickly eradicated using trapping and Talon 20P poison applied to the island in May 1996 (McKinley 1999). Tracking tunnel data from 1992 and 1993 confirmed the absence of mice on Mou Waho prior to their detection in 1995, and no mice have been detected since July 1996 (McKinley 1999).

### 2.2 Field Collection

Fieldwork was conducted under permit from the New Zealand Department of Conservation (DOC). Wētā were collected between 30 November and 1 December 2022 by boat. On weka‐free Mou Tapu, individuals were sampled from three sites: one at lake level on the south shore, one on the west side of the island, ~100 m above lake level, and one on the north shore, where wētā were abundant among shoreline rocks. On weka‐inhabited Mou Waho, wētā were more difficult to locate and were only found under very large rocks at the highest parts of the island despite searches at multiple sites around the island. In total, 19 males (9 intermediate, 9 melanic, 1 yellow) and 20 females (7 intermediate, 6 melanic, 7 yellow) were collected from Mou Tapu, and 17 males (11 intermediate, 4 melanic, 2 yellow) and 14 females (3 intermediate, 3 melanic, 8 yellow) from Mou Waho. Wētā were transported in individual ventilated containers (10.8 cm by 4.5 cm) to the Department of Zoology at the University of Otago.

### 2.3 Laboratory Housing

Upon arrival, wētā were housed in an animal containment facility with a controlled artificial light and climate regime. The day/night cycle was set to 14:10 h with a 1 h dawn/dusk ramp, and a reverse light cycle (dark at 14:00) to facilitate phenotyping. Temperature was maintained at 14°C during the day and 8°C at night, corresponding to the average summer conditions at the time of collection. Male–female pairs from the same location were housed in plastic enclosures (L 26 cm, W 14.5 cm, H 18 cm), containing a tile refuge (L 20 cm, W 10 cm, H 2.5 cm), a flax flower stem refuge, food and water dishes, and a leafy branch of coprosma (*Coprosma robusta*). Carrot was supplied ad libitum and replenished as needed. Containers were sprayed every 2 days to maintain humidity, and frass was removed weekly.

Five wētā died in captivity. Following phenotyping, the remaining wētā were returned to their respective islands on 20 December, ensuring that the wētā were well hidden under rocks on Mou Waho to prevent predation by weka that were nearby.

### 2.4 Morphological Data

We measured head length, head width, mandible length, pronotum length, right tibia length, and right femur length for all wētā using digital callipers (Kinchrome digital vernier calliper, no. 2313). Ovipositor length was measured for females. Body mass was recorded with an electronic balance.

### 2.5 Behavioural Quantification

#### 2.5.1 Refuge‐Seeking, Activity and Exploration Assays

To quantify refuge‐seeking behaviour, activity, and exploration in individual wētā, we conducted a series of behavioural assays under controlled laboratory conditions. Behaviour was recorded with a Sony HDR‐CX110 camera (60 fps) mounted above four identical arenas (L 24 cm, W 24 cm, H 24 cm), allowing four individuals to be tested simultaneously in separate arenas (Figure S1). The arenas were lined externally with black plastic and internally with fresh white paper towels between trials (Parli et al. 2020; Kelly et al. 2023; Thompson et al. 2024). Arenas were cleaned with 70% ethanol after each trial.

For refuge‐seeking trials (9:00–12:00, light on), each arena contained two components: (1) a terracotta refuge (L 10 cm, W 7.6 cm, H 2.5 cm), placed against the centre of the bottom wall, and (2) an acclimation container positioned at the arena centre (Figure S1). Four wētā (one individual per arena; one male and one female from each island tested concurrently) were placed in acclimation containers (H 10 cm, D 8.5 cm, covered with black duct tape) at arena centres for five minutes. After removal of containers, see‐through plastic lids with ventilation holes were placed on top and trials were filmed for 20 min. The refuge had entry/exit points on each side. Videos were scored for latency to first entry into the refuge following removal of the acclimation container. Latency to first entry was quantified using EthoVision v15 (Noldus Information Technology).

For activity trials (dark phase, red light only), the same procedure was followed, except there was no refuge (open arena assay; Figure S1). After five minutes' acclimation, trials were filmed for 20 min under night mode. Video recordings were analysed using EthoVision, which tracked individual movement within the arena. Activity was quantified as total distance moved. We also quantified exploration by dividing the arena into nine equal zones and calculating the standard deviation of distances moved across zones, providing an index of spatial exploration rather than overall activity.

#### 2.5.2 Defensive Response Assays

To simulate a predator attack and assess defensive behaviour in wētā, we conducted poke‐test assays in controlled arenas. Defensive behaviour was tested in arenas (L 24 cm, W 24 cm, H 24 cm) using a glass rod (L 25.5 cm, D 0.6 cm). Individual wētā (one per trial) were randomly selected, placed in acclimation containers at the arena centre, and left undisturbed for five minutes before the assay began. The acclimation container was then removed and the wētā was gently prodded on the right side of the abdomen with the glass rod until a defensive response was elicited, following the ‘poke test’ method of Field and Glasgow (2001), adapted by Parli et al. (2020), and modified by Thompson et al. (2024) for *H. maori*. This assay simulates predator attack. The wētā were prodded for a maximum of 10 times, and both the number of prods required to elicit a response and the type of response were recorded. Defensive responses included behaviours such as fending, fleeing, and stridulation. Arenas and rods were cleaned with 70% ethanol between trials.

### 2.6 Statistical Analysis

All analyses were conducted in R v4.3.0 (R Core Development Team 2023). We used generalised linear models (GLMs) and linear models (lme4 package; (Bates et al. 2015)) to test the effects of location (Mou Waho vs. Mou Tapu), sex (male vs. female), and their interaction on morphological variables and behavioural traits (defensive aggression, refuge‐seeking, activity). Interactions between location and sex were non‐significant in all models, so they were removed from the analyses.

A principal component analysis (PCA) was used to examine variation in morphological traits and to assess differences between sex and location. The PCA showed that right hind femur length loaded strongly (52%) on the second principal component (PC2), which captured variation in structural body size and locomotor morphology (with high loadings also for weight and hind tibia length). On this basis—and consistent with previous work identifying femur length as the most reliable proxy for overall body size in wētā (Jamieson 2002; Koning and Jamieson 2001; Thompson et al. 2024; Wehi and Hicks 2010) – right hind femur length was included as a covariate in all behavioural models.

Appropriate error structures were used for different response variables. Continuous behavioural traits, including distance moved, exploration (standard deviation of movement), and latency, were log‐transformed and analysed using linear models with Gaussian error structure. The number of pokes required to elicit a defensive response was analysed using GLM with Poisson error structure. The binary defensive response variables (fled, rasp, gape, fend) were analysed using binomial GLMs. Data distributions and residuals of models were inspected to assess assumptions, normality, dispersion, and model fit.

## 3 Results

### 3.1 Morphological Differences

**FIGURE 2:**
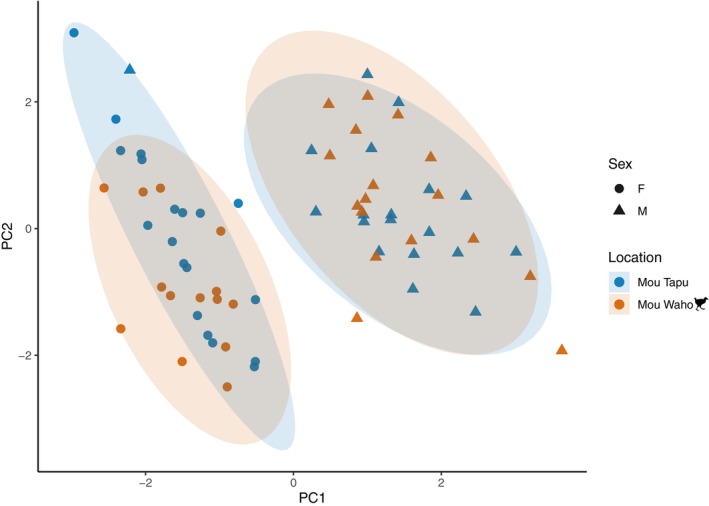
Principal component analysis (PCA) of morphological traits in *Hemideina maori* from Mou Tapu (*n*_female_ = 20, *n*_male_ = 19) and Mou Waho (*n*_female_ = 14, *n*_male_ = 17). Points represent individual wētā coloured by location and shaped by sex; ellipses indicate 95% confidence intervals. PC1 reflects head and mandible dimensions, whereas PC2 corresponds to structural body size (hind‐leg length and weight).

A principal component analysis (PCA) of morphological traits revealed no clear multivariate separation between wētā from Mou Tapu and Mou Waho (Figure 2). Individuals from both islands overlapped extensively along the first two principal components, which together described the majority of morphological variation. PC1 was primarily associated with head and mandible dimensions, while PC2 represented structural body size variation driven by hind‐leg length and weight.

Consistent with the PCA, linear models showed no significant differences in morphological traits between locations, with the exception of a non‐significant trend for right hind femur length to be greater in Mou Waho wētā than in those from weka‐free Mou Tapu (Table S1). In contrast, several morphological traits differed significantly between males and females, reflecting the expected sexual dimorphism in *H. maori*, including larger head and mandible dimensions in males (Table S1).

### 3.2 Refuge‐Seeking and Activity Behaviour

**FIGURE 3:**
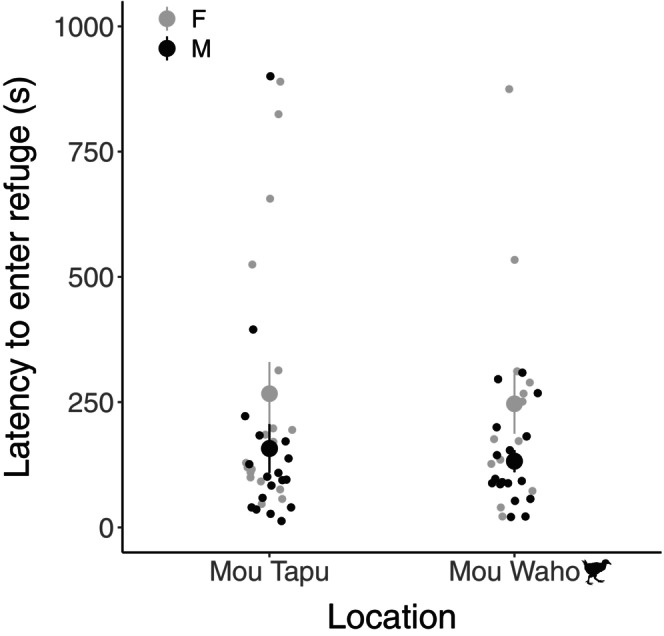
Latency to enter a refuge (s) for weka‐free Mou Tapu (*n*_female_ = 18, *n*_male_ = 18) and Mou Waho (*n*_female_ = 14, *n*_male_ = 17) *Hemideina maori* during 15‐min trials. If a wētā did not enter the refuge, latency was recorded as 900 s. Means for females (grey) and males (black) ± SEM are shown.

During the refuge seeking assay, latency to enter a refuge ranged from 0.22 to 900 s, with all but one wētā entering the refuge during the 15 min trials. Latencies to enter the refuge did not differ significantly between Mou Tapu and Mou Waho wētā (*b*_Location_ = −0.033 [−0.221, 0.160 CI]; *Z* = −0.332, *p* = 0.741; Figure 3), but there was a significant effect of sex, with females taking longer to enter the refuge than males (*b*_Sex_ = −0.218 [−0.409, −0.028 CI]; *Z* = −2.249, *p* = 0.028; Figure 3). Body size was not a significant covariate (*b*_Size_ = 0.083 [−0.015, 0.181 CI]; *Z* = 1.652, *p* = 0.104).

**FIGURE 4:**
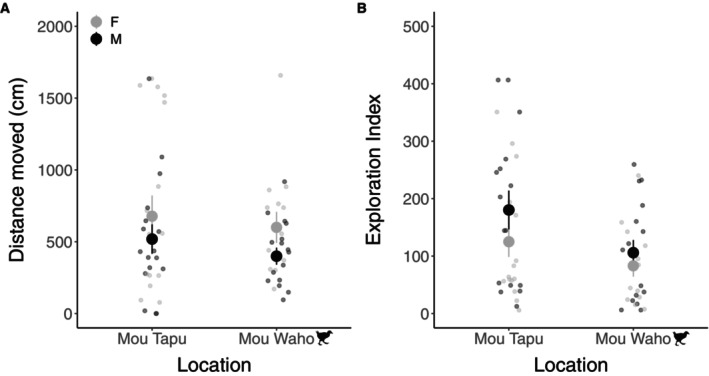
Total distance moved (A) and exploration (B) of Mou Tapu (*n*_female_ = 18, *n*_male_ = 18) and Mou Waho (*n*_female_ = 14, *n*_male_ = 17) *Hemideina maori* during 15‐min trials. Means for females (grey) and males (black) ± SEM are shown. Note that the data for panel A is plotted with 1 individual that was highly active removed.

Total distance moved, a measure of activity, did not differ significantly between Mou Tapu and Mou Waho wētā (*b*_Location_ = 0.010 [−0.414, 0.434 CI]; *t* = 0.049, *p* = 0.961; Figure 4A). There was no difference in activity between males and females (*b*_Sex_ = −0.023 [−0.442, 0.396 CI]; *t* = −0.108, *p* = 0.914), and no effect of size on activity (*b*_Size_ = 0.129 [−0.087, 0.346 CI]; *t* = 1.172, *p* = 0.246). Likewise, exploratory behaviour did not differ significantly between Mou Tapu and Mou Waho wētā (*b*_Location_ = −0.213 [−0.455, 0.029 CI]; *t* = −1.722, *p* = 0.091), though Mou Tapu wētā did tend to explore more (Figure 4B). There was no difference in activity between males and females (*b*_Sex_ = 0.010 [−0.138, 0.338 CI]; *t* = 0.820, *p* = 0.412), and no effect of size on activity (*b*_Size_ = −0.022 [−0.143, 0.099 CI]; *t* = −0.357, *p* = 0.723).

### 3.3 Defensive Behaviour

**FIGURE 5:**
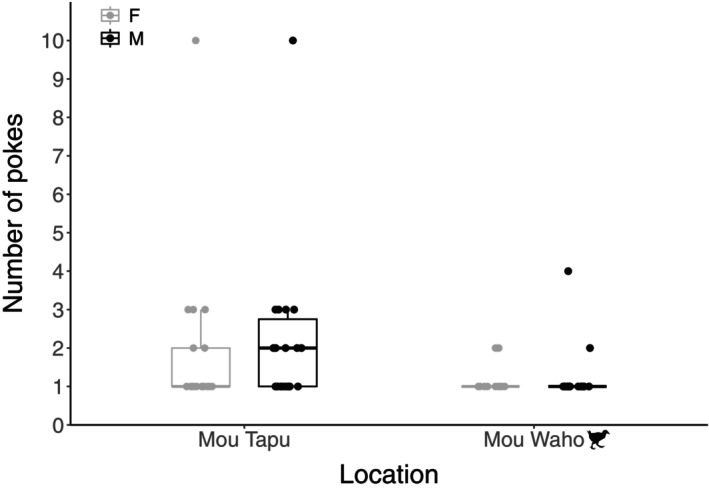
Number of pokes required to elicit a defensive response in Mou Tapu (*n*_female_ = 18, *n*_male_ = 18) and Mou Waho (*n*_female_ = 14, *n*_male_ = 17) *Hemideina maori*. Horizontal bars represent medians; lower and upper hinges correspond to the first and third quartiles. Whiskers extend to the largest and smallest values within 1.5 IQR (inter‐quartile range). Females are plotted in grey and males in black.

When we simulated a predator using the ‘poke’ test, defensive behaviours differed significantly between Mou Tapu and Mou Waho wētā (*b*_Location_ = −0.604 [−1.015, −0.210 CI]; *Z* = −2.05, *p* = 0.003; incidence rate ratio (IRR) = 0.55; Figure 5), with Mou Waho wētā requiring ~45% fewer pokes to elicit a defensive response than Mou Tapu individuals. Indeed, 83% of Mou Waho wētā responded to the first poke, whereas 43% of Mou Tapu wētā required more than one poke. Body size was a significant covariate, with larger individuals tending to be more defensive (*b*_size_ = 0.206 [0.017, 0.395 CI]; *Z* = 2.144, *p* = 0.032), but there was no difference between the sexes (*b*_sex_ = 0.157 [−0.214, 0.532 CI]; *Z* = 0.828, *p* = 0.408). Only three individuals required more than three pokes to respond, with the maximum being ten (2 wēta from weka‐free Mou Tapu). The most commonly observed defensive behaviours were fleeing and fending (raising hind legs). More males fended than females (*Z* = 2.53, *p* = 0.011), but there was no significant difference between Mou Tapu and Mou Waho wētā (*Z* = −1.009, *p* = 0.313). No other defensive behaviours (flee, rasp, mandible gape) were different between locations or sexes (Table S2).

## 4 Discussion

Our study provides a novel assessment of how exposure to an avian predator shapes antipredator behaviour in an invertebrate. Stone wētā (*Hemideina maori*) from Mou Waho, where ~200 weka are located (Department of Conservation, n.d.), showed significantly stronger defensive responsiveness than conspecifics from predator‐free Mou Tapu. However, we did not find any differences between the two populations in refuge use, general activity, or exploratory behaviour, but we observed marked differences in habitat use in the wild. These results highlight that antipredator behaviour comprises multiple partially independent components, some of which appear experience‐dependent while others remain stable across contrasting predation environments.

### 4.1 Predator Experience and Defensive Responsiveness

The most striking difference between populations was in the simulated attack assay: 83% of Mou Waho wētā responded after the first prod, whereas only 58% of Mou Tapu wētā responded after the first prod, with two individuals not responding to even 10 prods. This pattern is consistent with theoretical predictions of enhanced antipredator sensitivity in predator‐experienced populations (Carthey and Banks 2014; Cox and Lima 2006; Whitwell et al. 2012). Similar patterns of sensitivity have been shown in other New Zealand fauna. Robins rapidly lose vigilance after translocation to predator‐free environments (Muralidhar et al. 2019), and tree wētā on Nukuwaiata became markedly more relaxed after the eradication of kiore (Rufaut and Gibbs 2003). The slower, less reactive responses of Mou Tapu individuals suggest behavioural naivety associated with relaxed predation selection (Carthey and Banks 2014; Whitwell et al. 2012).

To explore the sensory basis of this heightened responsiveness, we conducted a pilot assay in which a taxidermy weka was moved toward individuals, but no observable response was elicited, consistent with the limited role of visual cues in nocturnal orthopterans (Field and Glasgow 2001).

Finally, the type of defensive behaviours expressed (e.g., fending, fleeing, rasping) did not differ between islands. Both populations retained the ancestral *Hemideina* antipredator repertoire (Field 2001; Field and Glasgow 2001; Thompson et al. 2024), but differed in the threshold at which these behaviours were initiated. This pattern mirrors other systems where naïve populations retain antipredator behaviours but deploy them only under high‐intensity cues (Ünlü et al. 2020; Wisenden et al. 1997).

### 4.2 Population Differences in Abundance and Habitat Use

Our observations during field collections revealed striking differences between islands. On Mou Tapu, wētā were abundant and distributed across shoreline and mid‐elevation rocky habitats. Indeed, most of our sampling took place at the shoreline, under rocks along the beach. In contrast, wētā on Mou Waho were extremely difficult to locate and were found only in artificial wētā boxes or under the largest rock slabs near the top of the island. We speculate that this distribution suggests that Mou Waho wētā likely experience predation pressure from weka, forcing them into more secure and higher elevation microhabitats that provide deeper protection.

Southern Alps geckos (*Woodworthia* ‘Southern Alps’) are common on both islands, and we observed especially high numbers on Mou Waho, frequently sheltering under rocks with wētā. Geckos are likely a predatory threat to nymphs and juveniles, but not to adults, and therefore are unlikely to explain the pronounced differences in adult abundance and distribution observed between islands.

Future work is required to confirm the role of weka predation; previous work on Mou Waho in 1999, prior to the re‐introduction of weka, reported *H. maori* were abundant and could be found in any sort of cover including on the side of cabbage trees under hanging dead leaves (E. Edwards, pers. commun.). We were not aware of this earlier finding during fieldwork and did not search beneath cabbage tree litter. More recent surveys (December 2025) that have included cabbage trees and their litter, however, have not proved fruitful. In any case, our repeated searches under rocks and tors—habitats typically used by stone wētā (Leisnham and Jamieson 2002)—indicate that the species is far less accessible or widespread on Mou Waho than on Mou Tapu, and less accessible and widespread than it was in 1999. Whether this reflects genuinely lower abundance, predator‐driven microhabitat shifts, or both remains an important consideration for interpreting behavioural differences.

A limitation of this study is that comparisons were conducted between only two island populations, with a single site representing each predator‐history treatment. Replicate systems matching these conditions are difficult to obtain in New Zealand, particularly for long‐isolated mammal‐free island populations of *H. maori* that differ primarily in weka presence. Although both islands are currently free of introduced mammalian predators, and records suggest that mice were only briefly present on Mou Waho prior to eradication (McKinley 1999), we cannot exclude the possibility that other site‐level differences contributed to the behavioural divergence observed between populations. Our findings should therefore be interpreted cautiously as evidence consistent with predator‐history effects, while recognising that other ecological differences between islands may also have contributed to the observed behavioural divergence.

### 4.3 Lack of Differences in Refuge‐Seeking and Activity

Despite pronounced differences in defensive responsiveness, refuge use and locomotor activity did not differ between populations. Several explanations are plausible. Refuge‐seeking may be a less plastic behaviour shaped by the species' natural ecology, and the presence of a guaranteed refuge in all laboratory trials may have overridden subtler population‐level differences. Open‐field locomotor assays also lack the structural complexity of natural tors, potentially limiting the ecological relevance of activity‐based antipredator measures (Kelly et al. 2023). Finally, predator experience may preferentially influence reactive responses to direct threats rather than proactive behaviours such as general activity or exploration (Blumstein 2006).

### 4.4 Sex and Size Effects

In contrast to our previous work with higher elevation *H. maori* (Thompson et al. 2024), larger wētā did not take longer to enter the refuge during the refuge‐seeking assay. Instead, females took longer to enter refuges than males. This pattern is consistent with sex‐specific behavioural strategies across *Hemideina*—males typically roam more widely in search of mates and may respond more quickly to disturbance (Koning and Jamieson 2001). It is also consistent with Farnworth et al. (2023), who reported sex differences in activity and foraging behaviour in Auckland tree wētā (*Hemideina thoracica*), with females spending more time feeding but less time moving, suggesting a potential trade‐off between foraging and antipredator responses. Although we detected no significant differences in morphology between locations, there was a weak trend for longer hind femurs in Mou Waho wētā, which could plausibly facilitate faster locomotor responses, but this pattern was not statistically supported. Body size did, however, predict defensiveness, consistent with previous findings that larger individuals are more likely to engage in active defence (Jamieson 2002; Wehi and Hicks 2010).

### 4.5 Implications for Conservation and Restoration

Our findings bear directly on invertebrate conservation and predator management in Aotearoa. Predator‐experienced populations such as those on Mou Waho may be better prepared for ecosystems where native predators persist or are reintroduced, whereas naïve populations like Mou Tapu could be disproportionately vulnerable if exposed to weka or other predators. At the same time, predator‐driven compression of habitat use—as suggested by Mou Waho's summit‐restricted wētā—could reduce population resilience by limiting access to food, mates, or favourable microclimates, particularly as climate change and conservation translocations alter the composition and intensity of predator communities, placing increasing importance on the plasticity of antipredator behaviour.

These results reinforce the need to incorporate behavioural assessments into conservation translocations, ecosanctuary planning, and predator reintroduction strategies (Gibbs 2009; Kelly et al. 2023). Behavioural naivety can rapidly elevate predation mortality, and identifying populations with reduced antipredator responsiveness may help managers mitigate risk.

## 5 Conclusion

Predator experience shapes key aspects of antipredator behaviour in stone wētā, with weka‐experienced Mou Waho individuals showing heightened reactive defence compared with weka‐free Mou Tapu wētā. Coupled with the restricted distribution of Mou Waho wētā in the field, our findings suggest ongoing predation pressure by weka—and highlight the importance of integrating behavioural ecology into invertebrate conservation planning. As invertebrates in global ecosystems continue to decline, and countries such as New Zealand need to prioritise conservation management and planning, understanding how prey species respond to both the removal and reintroduction of predators will be crucial for safeguarding endemic invertebrate biodiversity.

## Author Contributions

**Sheri Johnson:** conceptualization (equal), data curation (lead), formal analysis (lead), investigation (equal), methodology (equal), project administration (lead), resources (lead), software (lead), supervision (equal), validation (lead), writing – original draft (lead), writing – review and editing (equal). **Luke Thompson:** formal analysis (supporting), investigation (equal), methodology (equal), writing – review and editing (supporting). **Hamish Doogan:** investigation (equal), methodology (equal), writing – review and editing (supporting). **Priscilla Wehi:** conceptualization (equal), investigation (supporting), methodology (equal), project administration (supporting), resources (supporting), supervision (equal), writing – original draft (supporting), writing – review and editing (equal).

## Conflicts of Interest

The authors declare no conflicts of interest.

## Supporting information


**Figure S1:** Phenotyping setup used for refuge‐seeking, activity, and exploration assays. The left image shows the four arenas set up for the refuge‐seeking assay, with wētā inside acclimation chambers. The top‐right image shows wētā following removal of the circular chambers, during quantification of latency to enter the refuge. The bottom‐right image shows wētā recorded under infrared conditions during activity and exploration assays.
**Table S1:** Effects of location and sex on stone wētā morphology. Estimates are model coefficients (±SE) from linear models. Location contrasts compare Mou Waho (weka‐present) to Mou Tapu (weka‐free). Significant effects (*p* < 0.05) are shown in bold.
**Table S2:** Effects of body size (right hind femur length), location, and sex on defensive behaviours in *Hemideina maori*. Estimates are model coefficients (±SE) from binomial GLMs. Location contrasts compare Mou Waho (weka‐present) to Mou Tapu (weka‐free). Significant effects (*p* < 0.05) are shown in bold.

## Data Availability

All data and code are available at https://doi.org/10.17605/OSF.IO/VYMPC.
