# Supplementary material for: Predator Experience Shapes Behaviour: Comparing Stone Wētā (Hemideina maori) Populations With and Without Weka ( Gallirallus australis hectori )
Source: Ecol Evol. 2026 Jun 29;16(7):e73907. doi: 10.1002/ece3.73907 (PMC13317349; doi:10.1002/ece3.73907)
Supplement: Supplementary file 1 — Figure S1: Phenotyping setup used for refuge‐seeking, activity, and exploration assays. The left image shows the four arenas set up for the refuge‐seeking assay, with wētā inside acclimation chambers. The top‐right image shows wētā following removal of the circular chambers, during quantification of latency to enter the refuge. The bottom‐right image shows wētā recorded under infrared conditions during activity and exploration assays. Table S1: Effects of location and sex on stone wētā morphology. Estimates are model coefficients (±SE) from linear models. Location contrasts compare Mou Waho (weka‐present) to Mou Tapu (weka‐free). Significant effects (p < 0.05) are shown in bold. Table S2: Effects of body size (right hind femur length), location, and sex on defensive behaviours in Hemideina maori. Estimates are model coefficients (±SE) from binomial GLMs. Location contrasts compare Mou Waho (weka‐present) to Mou Tapu (weka‐free). Significant effects (p < 0.05) are shown in bold. [file ECE3-16-e73907-s001.docx]

**Supplementary Material for Johnson et al., 2026.**

**Predator Experience Shapes Behaviour: Comparing Stone Wētā (Hemideina maori) Populations With and Without Weka (*Gallirallus australis hectori*)**


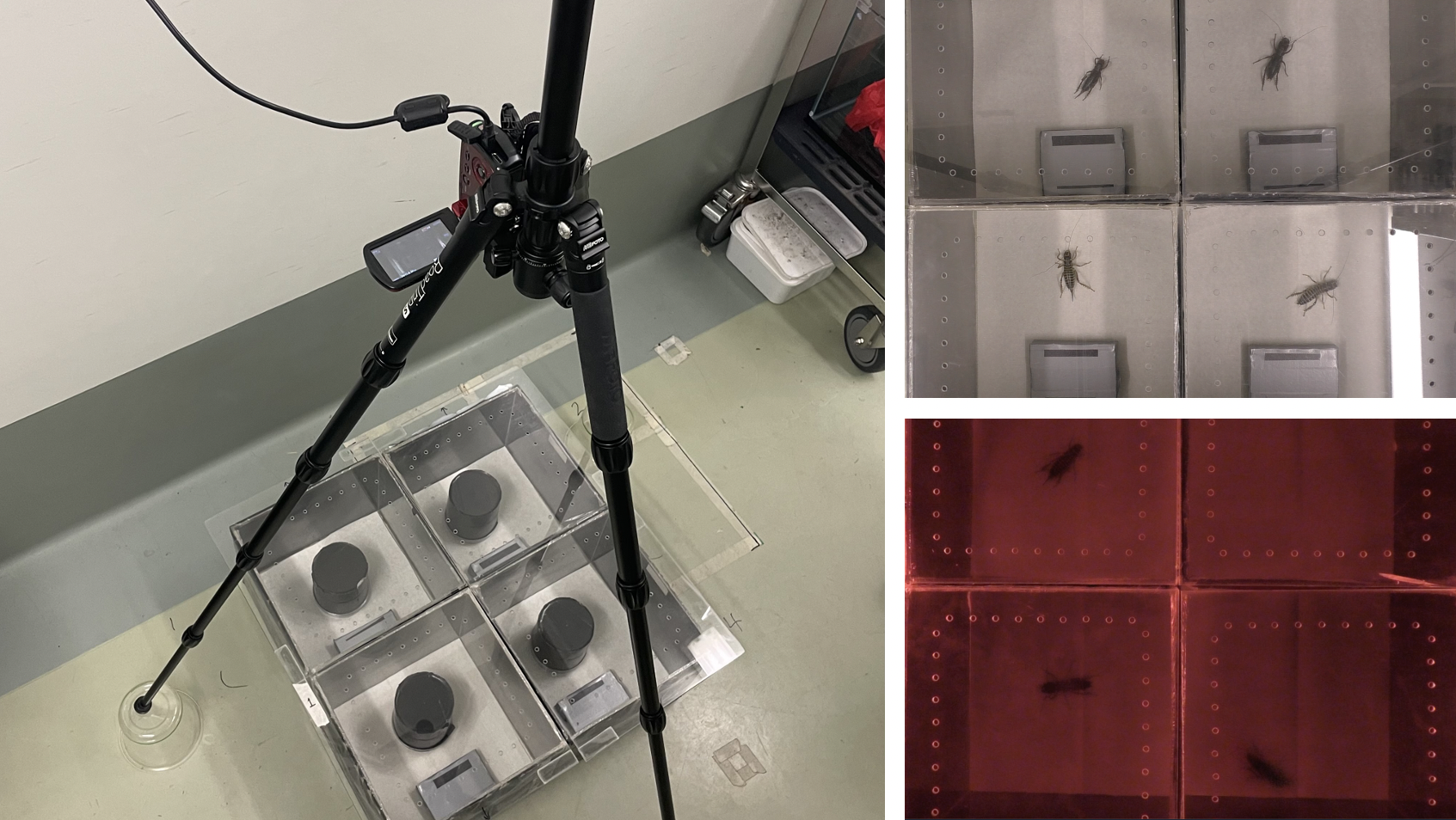


**Figure S1.** Phenotyping setup used for refuge-seeking, activity, and exploration assays. The left image shows the four arenas set up for the refuge-seeking assay, with wētā inside acclimation chambers. The top-right image shows wētā following removal of the circular chambers, during quantification of latency to enter the refuge. The bottom-right image shows wētā recorded under infrared conditions during activity and exploration assays.

**Table S1:** Effects of location and sex on stone wētā morphology. Estimates are model coefficients (± SE) from linear models. Location contrasts compare Mou Waho (weka-present) to Mou Tapu (weka-free). Significant effects (P < 0.05) are shown in bold.

| **Trait** | **Predictor** | **Estimate ± SE** | **Test statistic** | **P value** |
| --- | --- | --- | --- | --- |
| **Head width** | Location (Mou Waho vs Mou Tapu) | −0.03 ± 0.14 | −0.25 | 0.807 |
|  | Sex (Male vs Female) | 2.12 ± 0.14 | 15.24 | **<0.001** |
| **Head length** | Location (Mou Waho vs Mou Tapu) | 0.18 ± 0.33 | 0.55 | 0.584 |
|  | Sex (Male vs Female) | 4.58 ± 0.33 | 13.87 | **<0.001** |
| **Pronotum length** | Location (Mou Waho vs Mou Tapu) | −0.01 ± 0.09 | −0.12 | 0.905 |
|  | Sex (Male vs Female) | 0.04 ± 0.09 | 0.40 | 0.694 |
| **Right hind femur** | Location (Mou Waho vs Mou Tapu) | 0.49 ± 0.26 | 1.84 | 0.071 |
|  | Sex (Male vs Female) | −0.36 ± 0.26 | −1.36 | 0.180 |
| **Right tibia** | Location (Mou Waho vs Mou Tapu) | 0.20 ± 0.28 | 0.70 | 0.485 |
|  | Sex (Male vs Female) | −0.02 ± 0.28 | −0.09 | 0.932 |
| **Mandible length** | Location (Mou Waho vs Mou Tapu) | 0.15 ± 0.24 | 0.62 | 0.535 |
|  | Sex (Male vs Female) | 2.66 ± 0.24 | 10.93 | **<0.001** |
| **Body mass** | Location (Mou Waho vs Mou Tapu) | 0.08 ± 0.13 | 0.64 | 0.524 |
|  | Sex (Male vs Female) | −0.30 ± 0.12 | −2.37 | **0.021** |

**Table S2.** Effects of body size (right hind femur length), location, and sex on defensive behaviours in Hemideina maori. Estimates are model coefficients (± SE) from binomial GLMs. Location contrasts compare Mou Waho (weka-present) to Mou Tapu (weka-free). Significant effects (P < 0.05) are shown in bold.

| **Behaviour** | **Predictor** | **Estimate ± SE** | **z value** | **P value** |
| --- | --- | --- | --- | --- |
| Fleeing | Body size | −0.30 ± 0.29 | −1.04 | 0.299 |
|  | Location (Mou Waho vs Mou Tapu) | 0.86 ± 0.59 | 1.47 | 0.141 |
|  | Sex (Male vs Female) | −0.91 ± 0.58 | −1.58 | 0.114 |
| Rasping | Body size | −0.42 ± 0.38 | −1.12 | 0.262 |
|  | Location (Mou Waho vs Mou Tapu) | −0.18 ± 0.73 | −0.25 | 0.806 |
|  | Sex (Male vs Female) | 0.75 ± 0.76 | 0.98 | 0.326 |
| Mandible gaping | Body size | −2.32 ± 1.20 | −1.94 | 0.053 |
|  | Location (Mou Waho vs Mou Tapu) | −0.68 ± 1.36 | −0.50 | 0.620 |
|  | Sex (Male vs Female) | 19.55 ± 4079.80 | 0.01 | 0.996 |
| Fending | Body size | −0.08 ± 0.29 | −0.30 | 0.767 |
|  | Location (Mou Waho vs Mou Tapu) | −0.59 ± 0.58 | −1.01 | 0.313 |
|  | Sex (Male vs Female) | **1.55 ± 0.61** | 2.53 | **0.011** |
